# α-Amido Trifluoromethyl Xanthates: A New Class of RAFT/MADIX Agents

**DOI:** 10.3390/molecules29102174

**Published:** 2024-05-07

**Authors:** Mathias Destarac, Juliette Ruchmann-Sternchuss, Eric Van Gramberen, Xavier Vila, Samir Z. Zard

**Affiliations:** 1 Laboratoire Softmat, Université deToulouse, CNRS UMR 5623, Université Toulouse III-Paul Sabatier, 31062 Toulouse, France; 2 Rhodia Opérations, Centre de Recherches et Technologies d’Aubervilliers, 93308 Aubervilliers, France; juliette.ruchmann-sternchuss@saint-gobain.com (J.R.-S.); eric.van-gramberen@syensqo.com (E.V.G.); 3 Laboratoire de Synthèse Organique associé au C.N.R.S., UMR 7652, Ecole Polytechnique, 91128 Palaiseau, France; xavier.vila@cmail.cat

**Keywords:** xanthate, reactivity, RAFT polymerization, MADIX polymerization

## Abstract

Xanthates have long been described as poor RAFT/MADIX agents for styrene polymerization. Through the determination of chain transfer constants to xanthates, this work demonstrated beneficial capto-dative substituent effects for the leaving group of a new series of α-amido trifluoromethyl xanthates, with the best effect observed with trifluoroacetyl group. The previously observed Z-group activation with a *O*-trifluoroethyl group compared to the *O*-ethyl counterpart was quantitatively established with *C_ex_* = 2.7 (3–4 fold increase) using the SEC peak resolution method. This study further confirmed the advantageous incorporation of trifluoromethyl substituents to activate xanthates in radical chain transfer processes and contributed to identify the most reactive xanthate reported to date for RAFT/MADIX polymerization of styrene.

## 1. Introduction

**Scheme 1:**
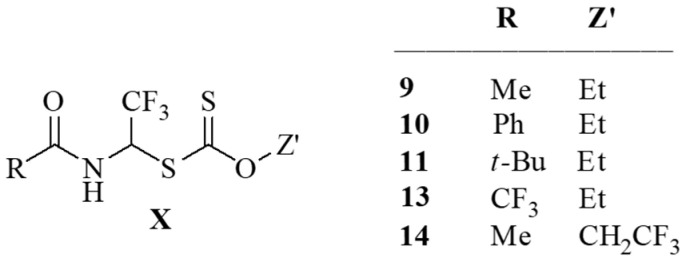
α-amido trifluoromethyl xanthates of the study.

Reversible addition-fragmentation chain transfer polymerization (RAFT), coined MADIX for Macromolecular Design via the Interchange of Xanthates (MADIX) for the specific use of xanthate chain transfer agents, recently celebrated its 25th anniversary [1,2,3,4]. Since its discovery, a significant amount of work has been performed on the design of new thiocarbonylthio chain transfer agents of the general structure R-S(C=S)Z [5]. Among them, dithiocarbonates (xanthates, Z=OZ’) have received increasing attention over the years and are today firmly established as best suited for the RAFT/MADIX polymerization of non-conjugated monomers [6,7,8]. Typical methods for xanthate synthesis are the reaction of a dithiocarbonate salt with an alkylating agent [9,10,11], thioacylation reactions [12,13], the ketoform reaction [14] and the radical substitution of a xanthogen disulfide [15]. The effectiveness of RAFT/MADIX agents depends on the nature of both activating and leaving groups, Z and R, respectively. For instance, we reported that *O*-ethyl xanthates (Z=OEt) with secondary leaving groups such as R = 1-phenylethyl and 1-(methoxycarbonyl)ethyl exhibit a moderate reactivity (C_tr_ = k_tr_/k_p_~0.5–1) in RAFT/MADIX polymerization of styrene (St) [16,17,18]. Later studies showed that the level of control over number-average molar masses (M_n_) and dispersities (Đ) could be markedly improved by selecting the electron-withdrawing CH_2_CF_3_ group as Z’ [10,17,18]. It appeared to us that there was still a great space for diversification and improvement in the selection of the leaving R group. In 2008, Zard et al. [19] described the use of α-acetamido trifluoromethyl xanthates as a powerful reagent for the introduction of geminal amido and trifluoromethyl groups through radical addition to olefins. Drawing on this work, we here report on the synthesis of a series of xanthates with α-amido trifluoromethyl leaving groups (Scheme 1) and the evaluation of their reactivity in RAFT/MADIX bulk polymerization of styrene. The choice of St was motivated by the moderate reactivity of xanthates to this monomer, thus allowing us to simply establish a structure–reactivity relationship via the determination of distinctive kinetic data.

## 2. Results and Discussion

### 2.1. Xanthate Synthesis

**Scheme 2:**
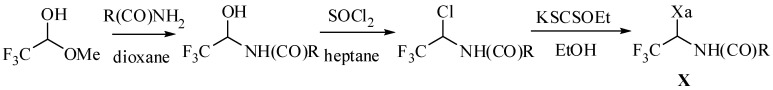
Synthetic strategy for α-amido trifluoromethyl xanthates. Xa = S(C=S)OEt.

The synthesis of the series of α-amido trifluoromethyl *O*-ethyl xanthates **9**–**12** was performed as follows (Scheme 2): the condensation between fluoral (trifluoroacetaldehyde methyl hemiacetal) and different amides afforded intermediate *N*-acyl hemiaminals, which in turn were converted to the corresponding chlorides, and these were eventually transformed to the desired xanthates via nucleophilic substitution with potassium ethylxanthogenate.

**Scheme 3:**
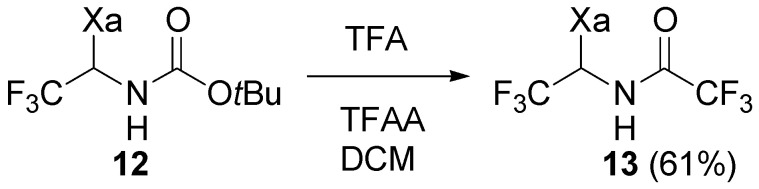
Synthesis of xanthate **13** (Xa = S(C=S)OEt).

Four groups of compounds were synthesized, with methyl, phenyl, *t*-butyl and *t-*butoxy R substituents corresponding to products **1** to **12** of Table 1. *t*-BuO was considered as a R group to cope with the failure of the direct condensation between trifluoroacetaldehyde hemiacetal and trifluoroacetamide. Thus, the series of products **4**, **8** and **12** was synthesized and the *t-*Boc group of xanthate **12** was eventually removed to introduce the trifluoroacetyl group and obtain xanthate **13** (Scheme 3).

Condensation between the methyl hemiacetal of fluoral and different amides afforded the desired alcohols in moderate yields. In the case of alcohol **4**, a longer reaction time was needed, generally 4 h as opposed to 1 h for alcohols **1** to **3**. The obtained alcohols were then transformed into the corresponding chlorides via reaction with SOCl_2_ in heptane. The reaction was fast and clean, the desired chlorides were obtained in good yields and generally no purification was furthermore needed. Finally, the conversion of these to the desired xanthates was carried out by a reaction with the potassium salt of ethylxanthic acid in ethanol, and the xanthates were obtained in good yields. As mentioned before, for the synthesis of trifluoroacetamido xanthate **13**, treatment of xanthate **12** with trifluoroacetic acid and trifluoroacetic anhydride in dichloromethane afforded the desired compound in 61% yield (Scheme 3).

Also, we synthesized xanthate **14** (Scheme 1) with the Z=OEt group being replaced with a 2,2,2-trifluoroethyl group, with R=Me. The commercially available 2,2,2-trifluoroethanol was treated with sodium hydride in the presence of an excess of carbon disulfide in DMF at −40 °C, followed by the addition of the corresponding alkyl halide. The temperature was an important factor because the reaction of sodium trifluoroethoxide with carbon disulfide leading to the desired xanthate salt is quite reversible, due to the high inductive effect of the trifluoromethyl group. Carrying the reaction at higher temperatures favors the reverse process, and under these conditions, the only isolated product after addition of the electrophile is the ether resulting from the substitution of the chloride by the trifluoroethoxide anion. In contrast, the formation of the xanthate proceeded in good yield at the reported low temperature.

### 2.2. Structure–Reactivity Relationship of α-Amido Trifluoromethyl Xanthates

Self-initiated bulk polymerization of St was carried out at 110 °C in sealed tubes under vacuum. The transfer constants to xanthates C_tr_ were determined from both the Mayo method [20] and Equation (1) derived from the kinetic model established by Müller et al. [21] for living polymerization systems exhibiting slow degenerative transfer:

(1) $$Ln\left(1-\frac{DP_{n,th}}{DP_{n,calc}}\right)=C_{tr}Ln\left(1-conv\right)$$

where DP_n,th_ is the theoretical number-average degree of polymerization (equal to ([M]_0_/[X]_0_).conv. for an instantaneous xanthate consumption, where conv. represents the fractional monomer conversion) and DP_n,calc_ is the calculated DP_n_ when X was not fully reacted (DP_n,calc_ = conv.[M]_0_/Δ[X]). DP_n,calc_ can be assimilated to DP_n,SEC_ provided that the contribution of initiator-derived chains is negligible. With this method, C_tr_ may be slightly overestimated when the contribution of initiation (therefore termination) is such that 2fΔ[I_2_] << Δ[X] cannot be applied. As for the Mayo method, based on the Expression (2) giving the evolution of [X]/[M] during polymerization, C_tr_ values are only valid at low monomer conversions and low C_tr_ and tend to be underestimated at greater conversions and C_tr_.

(2) $$\frac{\left[X\right]}{\left[M\right]}=\frac{{\left[X\right]}_{0}}{{\left[M\right]}_{0}}{\left(1-conv\right)}^{C_{tr}-1}$$

Thus, it can be propounded that the real C_tr_ value lies between those determined by the two chosen methods.

**Figure 1:**
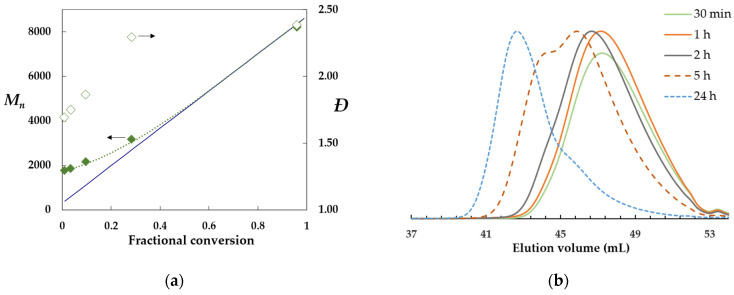
(**a**) Evolution of M_n_ (closed symbol) and Đ (open symbol) for **13**-mediated RAFT/MADIX polymerization of St in bulk at 110 °C. Solid line: theoretical M_n_. The dashed line is a guide to the eye. (**b**) Overlay of SEC traces.

The C_tr_ values are listed in Table 2. The experimental data used for plotting Mayo regressions for xanthates **9**, **10**, **11** and **13** are gathered in Table S1. The corresponding Mayo plots can be found in Figures S1–S4. The data used for the calculation of C_tr_ for **13** DP_n,calc_ and **14** from Equation (1) are available in Table S2. C_tr_ values are moderate (comprised between 2 and 3) and nearly equal for xanthates **9**, **10** and **11**, reflecting a similar stabilizing effect of methyl, phenyl and *t*-butyl R substituents on the leaving group radical. The Mayo method and the one derived from Müller’s kinetic model gave similar results. It is worth mentioning that these values are ~3-times greater than those previously reported for *O*-ethyl xanthates with classically used 1-phenylethyl and 1-(methoxycarbonyl)ethyl leaving groups [16,17]. However, at low conversions (~5–10%), M_n_ values are much higher than those expected for a controlled RAFT/MADIX process, and dispersities are in the range 1.85–2.35 (Table S1). This observation is typical of RAFT/MADIX systems, for which both C_tr_ (for starting RAFT/MADIX agent) and C_ex_ (for macro-RAFT/MADIX agent PSt-X, C_ex_ = k_ex_/k_p_, with k_ex_ the exchange rate constant) are low. This is supported by results of Catala et al., who reported a C_ex_ value of 0.7–0.8 for the St/PSt-S(C=S)OEt system [22]. In contrast, the trifluoromethyl R group in **13** further increases the electrophilic character of the leaving group radical and facilitates the fragmentation step, resulting in a greater C_tr_ equal to 6.1. It is worth mentioning that the Mayo regression for **13** was plotted with St conversions of about 1% in order to minimize the error in the determination of C_tr_. Using DP_n_,_calc_ values extracted from Figure 1 or Table S2, the same C_tr_ value of 6.1 was obtained from Equation (1). On Figure 1, it can be seen that as a consequence of the high C_tr_ value, the evolution of M_n_ with conversion approaches linearity. Dispersities are high and increase from 1.69 to 2.39 during polymerization. This can be explained by a slow equilibrium between dormant PSt-**13** chains and macroradicals (C_ex_~0.7–0.8 for *O*-ethyl xanthates) [22], responsible for the presence of multimodality in molar mass distributions (Figure 1b).

**Figure 2:**
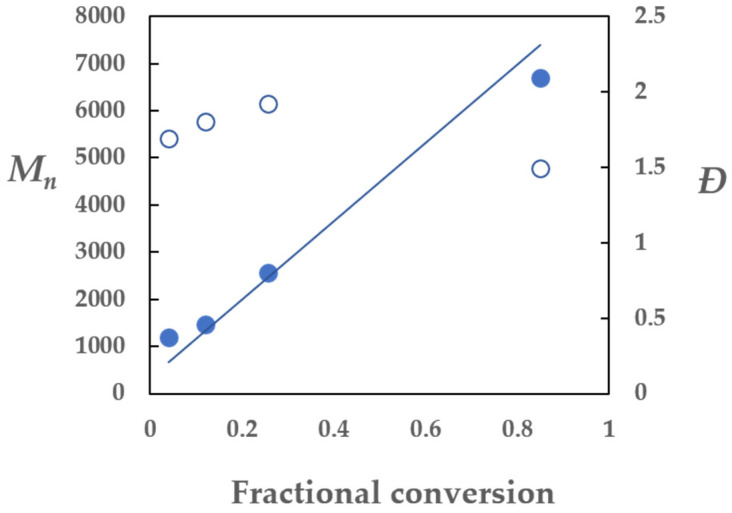
Evolution of M_n_ (closed symbol) and Đ (open symbol) for **X14**-mediated RAFT/MADIX polymerization of St in bulk at 110 °C.

A similar behavior was reported by our group for *S*-cyanoisopropyl *O*-ethyl xanthate, for which a C_tr_ of 6.8 close to that of **13** had been determined [16]. Due to a capto-dative stabilizing effect for the leaving group radical, this series of α-amido trifluoromethyl groups represents the best secondary leaving groups ever reported for *O*-ethyl RAFT/MADIX agents. The carbon–sulfur bond is weakened by an anomeric type effect due to the lone pair on the nitrogen (a lone pair–σ* interaction), which may explain the efficiency of these xanthates. In agreement with some of our earlier studies [10,17,18], the reactivity of the xanthate was greatly increased by substituting the *O*-ethyl Z group of **13** for a *O*-CH_2_CF_3_ in **14**. A C_tr_ of 15 was determined from Equation (1) (Table S2). This was confirmed with the observed good control of M_n_ with conversion (Figure 2). At 85% St conversion, Đ equals 1.49 (Figure 2), which reflects a faster interchain transfer compared to *O*-ethyl analogs. This increase in reactivity owing to the trifluoroethyl Z group is quantified in the next section through the determination of C_ex_ for xanthate **14**.

### 2.3. Determination of C_ex_ for Z=OCH_2_CF_3_

**Figure 3:**
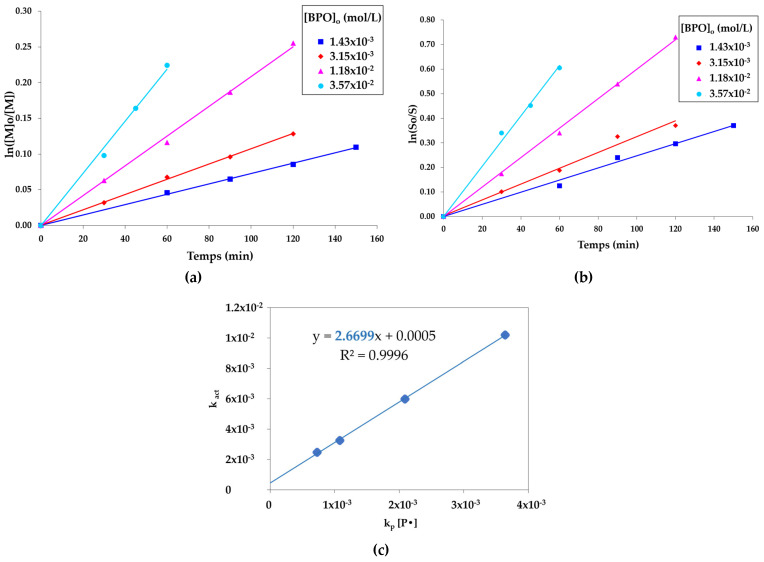
Determination of C_ex_ for PSt−**14** at 80 °C. [PSt−**14**]_0_ = 1.8 × 10^−2^ mol/L. [BPO]_0_ as indicated in the figure. (**a**) Plot of Ln([M]_0_/[M]) vs. time. (**b**) Plot of Ln([S]_0_/[S]) vs. time. (**c**) Plot of k_act_ vs. k_p_[P•].

We applied the SEC-based peak resolution method developed by Fukuda et al. [23] to determine the activation rate constant k_act_ for PSt-S(C=S)OCH_2_CF_3_ (PSt-**14**) in St polymerization. A low molar mass PSt-**14** was synthesized (M_n_ = 1850 g/mol, Đ = 1.3). The fraction of dead chains F_dead_ was estimated to 6.4% from a chain extension test and treatment of the resulting SEC trace (see Appendix A for details). F_dead_ was taken into account in the data treatment for the determination of k_act_, as dead chains do not contribute to PSt-**14** activation. A series of polymerizations were carried out at 80 °C with [PSt-**14**]_0_ = 1.8.10^−2^ mol/l and varying values of [BPO]_0_ (Figure 3a,b). Figure 3a gives the first-order plots of the monomer concentration [M], hence [P•] for each [BPO]_0_. Here St conversion was directly determined from SEC traces, which directly give the concentration of formed PSt. The resolution of the bimodal SEC curves of each raw polymerization product of the series allowed for the determination of the activation rate coefficient k_act_ from the slope of ln(S/S_0_) vs. time (Figure 3b and Figure S5) [23].

ln(S_0_/S) = k_act_t (3)

The plot of k_act_ = k_d_ + k_ex_[P•] against [P•] makes a straight line with its intercept at [P•] = 0 and slope giving k_d_ and k_ex_, respectively. The data points from Figure 3a,b give a straight line with k_d_~0 and *C_ex_*= 2.67 (Figure 3c). These results are in agreement with the experimental Đ values of the present and past studies [10,17] (model predictions give Đ ~1 + 1/C_ex_ at the end of the polymerization) [21].

## 3. Materials and Methods

### 3.1. Materials

St was purchased from Aldrich. It was distilled under vacuum and stored over CaH_2_ prior to any use. All the reactants that took part in the synthesis of the xanthates were purchased from Aldrich and used as received.

### 3.2. Synthesis of the α-Amido Trifluoromethyl O-ethyl Xanthates

General method for the synthesis of trifluoromethyl alcohols. To a solution of fluoral (1 equiv.) in dioxane (1.5 mL/mmol), the corresponding amide (1 equiv.) was added and the mixture was heated at reflux temperature for one hour. After that time, the reaction mixture was concentrated, and the residue was purified by the corresponding method.

The synthesis protocols of compounds **1** [19], **2** [24] and **4** [24] were already reported in the literature.

*2,2-Dimethyl-N-(2,2,2-trifluoro-1-hydroxyethyl)propionamide* **3**. Purified by flash column chromatography (petrol:EtOAc 1:1), 40%, white solid. mp: 108–110 °C. IR (CCl_4_) ν_max_/cm^−1^ 3350 (NH), 1689 (CO). ^1^H-NMR (CD_3_OD) δ 1.20 (s, 9 H, 3 CH_3_), 4.89 (bs, 2 H, OH and NH), 5.78 (q, *J* = 5.6 Hz, 1 H, CH). ^13^C-NMR (CD_3_OD) δ 27.6 (3 CH_3_), 40.0 (C), 72.5 (q, *J* = 35.3 Hz, CH), 124.8 (q, *J* = 279.9 Hz, CF_3_), 181.4 (CO). MS *m*/*z* (CI) 200 (MH^+^), 217 (MNH_4_^+^).

General method for the synthesis of trifluoromethyl chlorides. To a suspension of alcohol (1 equiv.) in heptane (2 mL/mmol), SOCl_2_ (1.1 equiv.) was added and the resulting mixture was heated to 85 ºC. The reaction mixture turned from cloudy to transparent. It was stirred for a further 15 min and then cooled down. When it was cold, a solid precipitated. It was filtered and washed with cold petrol ether, thus obtaining the desired chloride, which was used in the next step without further purification.

Compounds **5** [19] and **6** [24] were already reported in the literature.

*N-(1-Chloro-2,2,2-trifluoroethyl)-2,2-dimethylpropionamide* **7**. No further purification was needed, white solid. mp: 81 °C. IR (CCl_4_) ν_max_/cm^−1^ 3351 (NH), 1652 (CO). ^1^H-NMR (CDCl_3_) δ 1.21 (s, 9 H, 3 CH_3_), 6.31–6.65 (m, 1 H, CH), 6.64 (d, *J* = 9.6 Hz, 1 H, NH). ^13^C-NMR (CDCl_3_) δ 26.8 (3 CH_3_), 39.0 (C), 61.1 (q, *J* = 38 Hz, CH), 121.8 (q, *J* = 277 Hz, CF_3_), 177.9 (CO). MS *m*/*z* (CI) 217 (MH^+^).

*(1-Chloro-2,2,2-trifluoroethyl)carbamic acid tert-butyl ester* **8**. No further purification was needed, white solid. mp: 86–87 °C. ^1^H-NMR (CDCl_3_) δ 1.49 (s, 9 H, 3 CH_3_), 5.20–5.45 (m, 1 H, NH), 6.05–6.15 (m, 1 H, CH). IR (CCl_4_) ν_max_/cm^−1^ 3440 (NH), 1735 (CO), 1503 (NH), 1200 (C-O), 1156 (C-N).

General method for the synthesis of trifluoromethyl xanthates. To a solution of chloride (1 equiv.) in absolute EtOH (4 mL/mmol) cooled to 0 °C, potassium ethylxanthogenate (1.2 equiv.) was added and the mixture was stirred at room temperature for 2 h. After that time, the reaction mixture was concentrated, the residue redissolved in Et_2_O and washed with H_2_O. The organic phase was dried and evaporated, and the residue purified by the corresponding method.

*Dithiocarbonic acid (1-acetylamino-2,2,2-trifluoroethyl) ester ethyl ester* **9**. Purified by crystallization (petrol:EtOAc), 87%, white solid. mp: 76–78 °C. IR (CCl_4_) ν_max_/cm^−1^ 3441 (NH), 1713 (CO), 1491 (NH), 1236 (C-O), 1048 (C=S). ^1^H-NMR (CDCl_3_) δ 1.43 (t, *J* = 7.2 Hz, 3 H, CH_3_), 2.08 (s, 3 H, CH_3_), 4.68 (q, *J* = 7.2 Hz, 2 H, OCH_2_), 6.58 (dq, *J* = 8, 9.6 Hz, 1 H, CH), 7.04 (d, *J* = 9.6 Hz, 1 H, NH). ^13^C-NMR (CDCl_3_) δ 13.5 (CH_3_), 22.8 (CH_3_ NAc), 57.7 (q, *J* =34.3 Hz, CH), 71.3 (OCH_2_), 123.3 (q, *J* = 279 Hz, CF_3_), 169.6 (CO), 206.8 (CS). MS *m*/*z* (CI) 262 (MH^+^), 279 (MNH_4_^+^).

*Dithiocarbonic acid (1-benzoylamino-2,2,2-trifluoroethyl) ester ethyl ester* **10**. Purified by flash column chromatography (petrol:EtOAc 9:1), 65%, white solid. mp: 106–108 °C. IR (CCl_4_) ν_max_/cm^−1^ 3447 (NH), 1694 (CO), 1507 (NH), 1242 (C-O), 1046 (C=S). ^1^H-NMR (CDCl_3_) δ 1.41 (t, *J* = 7.2 Hz, 3 H, CH_3_), 4.65 and 4.66 (2 q, *J* = 7.2 Hz each, 1 H each, OCH_2_), 6.75–6.85 (m, 1 H, CH), 7.40–7.90 (m, 5 H, ArH). ^13^C-NMR (CDCl_3_) δ 13.5 (CH_3_), 58.1 (q, *J* = 34.4 Hz, CH), 71.2 (OCH_2_), 123.4 (q, *J* = 279 Hz, CF_3_), 127.6 (CH Ar), 128.6 (CH Ar), 132.3 (C Ar), 132.5 (CH Ar), 166.6 (CO), 206.8 (CS). MS *m*/*z* (CI) 324 (MH^+^), 341 (MNH_4_^+^).

*Dithiocarbonic acid [1-(2,2-dimethylpropionylamino)-2,2,2-trifluoroethyl] ester ethyl ester* **11**. Purified by crystallization (petrol:EtOAc), 79%, white solid. mp: 94–95 °C. IR (CCl_4_) ν_max_/cm^−1^ 3457 (NH), 1699 (CO), 1488 (NH), 1245 (C-O), 1047 (C=S). ^1^H-NMR (CDCl_3_) δ 1.20 (s, 9 H, 3 CH_3_), 1.42 (t, *J* = 7.2 Hz, 3 H, CH_3_ xanthate), 4.60–4.72 (m, 2 H, OCH_2_), 6.43 (d, *J* = 9.6 Hz, 1 H, NH), 6.53 (dq, *J* = 7.6, 9.6 Hz, 1 H, CH). ^13^C-NMR (CDCl_3_) δ 13.5 (CH_3_), 27.0 (3 CH_3_), 38.9 (C), 57.7 (q, *J* = 34.3 Hz, CH), 71.1 (OCH_2_), 123.4 (q, *J* = 280 Hz, CF_3_), 177.4 (CO), 206.9 (CS). MS *m*/*z* (CI) 304 (MH^+^), 321 (MNH_4_^+^).

*Dithiocarbonic acid (1-tert-butoxycarbonylamino-2,2,2-trifluoroethyl) ester ethyl este*r **12**. Purified by flash column chromatography (petrol:EtOAc 95:5), white solid. mp: 75–76 °C. IR (CCl_4_) ν_max_/cm^−1^ 3442 (NH), 1737 (CO), 1495 (NH), 1237 (C-O), 1196 (C-O), 1155 (C-N), 1048 (C=S). ^1^H-NMR (CDCl_3_) δ 1.46 (t, *J* = 7.2 Hz, 3 H, CH_3_ xanthate), 1.47 (s, 9 H, 3 CH_3_), 4.65–4.75 (m, 2 H, OCH_2_), 5.16 (d, *J* = 8.8 Hz, 1 H, NH), 6.22–6.34 (m, 1 H, CH). ^13^C-NMR (CDCl_3_) δ 13.5 (CH_3_ xanthate), 28.0 (3 CH_3_), 60.0 (q, *J* = 34 Hz, CH), 71.0 (OCH_2_), 81.7 (C Boc), 123.4 (q, *J* = 278.8 Hz, CF_3_), 153.4 (CO), 207.1 (CS). MS *m*/*z* 320 (MH^+^), 337 (MNH_4_^+^).

*Dithiocarbonic acid ethyl ester [2,2,2-trifluoro-1-(2,2,2-trifluoro-acetylamino)ethyl] ester* **13**. To a solution of xanthate **12** (519 mg, 1.63 mmol) in CH_2_Cl_2_ (2 mL) cooled to 0 ºC, trifluoroacetic anhydride (6.9 mL, 48.9 mmol) and trifluoroacetic acid (3.8 mL, 48.9 mmol) were added and the mixture was stirred at room temperature for 3 h. After that time, the reaction mixture was concentrated, the residue was purified by flash column chromatography (petrol:EtOAc 96:4) and the desired trifluoroacetamide **13** was obtained as a brown solid (314 mg, 61%). mp: 55–56 °C. IR (CCl_4_) ν_max_/cm^−1^ 3426 (NH), 1755 (CO), 1520 (NH), 1227 (C-O), 1207 (C-O), 1157 (C-N), 1045 (C=S). ^1^H-NMR (CDCl_3_) δ 1.46 (t, *J* = 7.2 Hz, 3 H, CH_3_), 4.70 and 4.71 (2 q, *J* = 7.2 Hz each, 1 H each, OCH_2_), 6.47 (dq, *J* = 7.2, 9.6 Hz, 1 H, CH), 6.84 (d, *J* = 9.2 Hz, 1 H, NH). ^13^C-NMR (CDCl_3_) δ 13.4 (CH_3_), 58.2 (q, *J* = 17.5 Hz, CH), 71.9 (OCH_2_), 115.3 (q, *J* = 285.5 Hz, CF_3_), 122.7 (q, *J* = 279.3 Hz, CF_3_), 156.9 (q, *J* = 39.1 Hz, CO), 205.1 (CS).

*Synthesis of the O-trifluoroethyl xanthate* **14**. To a solution of 2,2,2-trifluorethanol (1 equiv.) in DMF (2 mL/mmol) cooled to −40 °C, CS_2_ (20 equiv.) and NaH (1.1 equiv.) were added and the mixture was stirred at this temperature for 2 h. Then, the corresponding alkyl halide (1.1 equiv.) was added and stirred at the same temperature for a further hour. After that time, the reaction mixture was diluted with EtOAc and washed four times with H_2_O. The organic phase was dried and evaporated, and the residue was purified by flash column chromatography.

*Dithiocarbonic acid (1-acetylamino-2,2,2-trifluoroethyl) ester (2,2,2-trifluoroethyl) ester* **14**. Purified by flash column chromatography (petrol:EtOAc 7:3), 91%, white solid. mp: 78–79 °C. IR (CCl_4_) ν_max_/cm^−1^ 3441 (NH), 1718 (CO), 1277 (C-O), 1202 (C-O), 1090 (C=S). ^1^H-NMR (CDCl_3_) δ 2.09 (s, 3 H, CH_3_), 4.80–5.00 (m, 2 H, OCH_2_), 6.56 (qd, *J* = 7.6, 9.6 Hz, 1 H, CH), 6.97 (d, *J* = 9.6 Hz, 1 H, NH). ^13^C-NMR (CDCl_3_) δ 22.7 (CH_3_), 59.2 (q, *J* = 34.6 Hz, CH), 68.0 (q, *J* = 37 Hz, OCH_2_), 123.2 (q, *J* = 278.8 Hz, CF_3_), 122.3 (q, *J* = 275.7 Hz, CF_3_), 169.7 (CO), 206.1 (CS). MS *m*/*z* (CI) 316 (MH^+^), 333 (MNH_4_^+^).

Polymerizations. Bulk polymerization of styrene was carried out at 110 °C in sealed tubes after degassing by three pump–freeze–thaw cycles. For the determination of C_tr_ values by the Mayo method, polymerizations were run at different initial xanthate concentrations and were stopped at low St conversion (<10%). When Equation (1) was used with xanthates **9**, **10** and **11**, *C_tr_* was determined as the average of all values calculated for different [X]_0_. For xanthates **13** and **14**, *C_tr_* was calculated from Equation (1) based on one single [X]_0_ from different conversion points. Conversion was determined by gravimetry.

### 3.3. Characterization

NMR spectra were recorded in CDCl3 using a Bruker AMX400 operating at 400 MHz for 1 H and 100 MHz for 13 C. The chemical shifts are expressed in parts per million (ppm) referenced to residual chloroform. 1 H NMR data are reported as follows: δ, chemical shift; multiplicity (recorded as: s, singlet; bs, broad singlet; d, doublet; t, triplet; q, quadruplet; dq, double quadruplet; m, multiplet), coupling constants (J are given in Hertz, Hz) and integration. Infrared absorption spectra were recorded as a solution in CCl4 with a Perkin-Elmer 1600 Fourier Transform Spectrophotometer. Mass spectra were recorded with an HP 5989B mass spectrometer via direct introduction for chemical positive ionization (CI) using ammonia as the reagent gas. Melting points were determined by Reichert microscope apparatus and were uncorrected. HRMS were performed on JEOL JMS-GcMate II, GC/MS system spectrometer.

Number-average molar masses (M_n_) and dispersities (Đ) were measured by size-exclusion chromatography (SEC), using the following Phenogel columns: guard, linear, 1000 Å and 100 Å (eluent: THF (1 mL/mn). A differential refractive index detector was used, and molecular weights were calculated based on PSt standards.

## 4. Conclusions

To conclude, a new series of xanthate RAFT/MADIX agents bearing α-amido trifluoromethyl leaving groups and exhibiting very good ability for fragmentation was kinetically investigated for styrene polymerization. Quantitative measurements of both C_tr_ for xanthates and C_ex_ faithfully supported the experimental observations for molar masses and dispersities. For xanthate **14**, the trifluoroacetyl group gave the best ability for leaving group fragmentation, with the best activation brought by the *O*-trifluoroethyl Z group. If we consider that xanthates are commonly known to be poor RAFT/MADIX agents for styrene, this study further established the advantageous incorporation of trifluoroalkyl substituents to activate xanthates in radical chain transfer processes.

## Figures and Tables

**Table 1:** Series of alcohol and chloride precursors and xanthate products.

R	Alcohol (%)	Chloride (%)	Xanthate (%)
Me	**1** ^a^ (54) ^b^	**5** (91)	**9** (87)
Bz	**2** (64)	**6** (71)	**10** (65)
tBu	**3** (40)	**7** (89)	**11** (79)
tBuO	**4** (36)	**8** (65)	**12** (52)

^a^ Product number; ^b^ yield.

**Table 2:** Transfer constants to xanthates.

X	C_tr_
**9**	2.1 ^a^ (3.1) ^b^
**10**	2.5 (3.2)
**11**	2.2 (2.9)
**13**	6.1 (6.1)
**14**	(15)

^a^ Determined from the Mayo plot. ^b^ From Equation (1).

**Figure A1:**
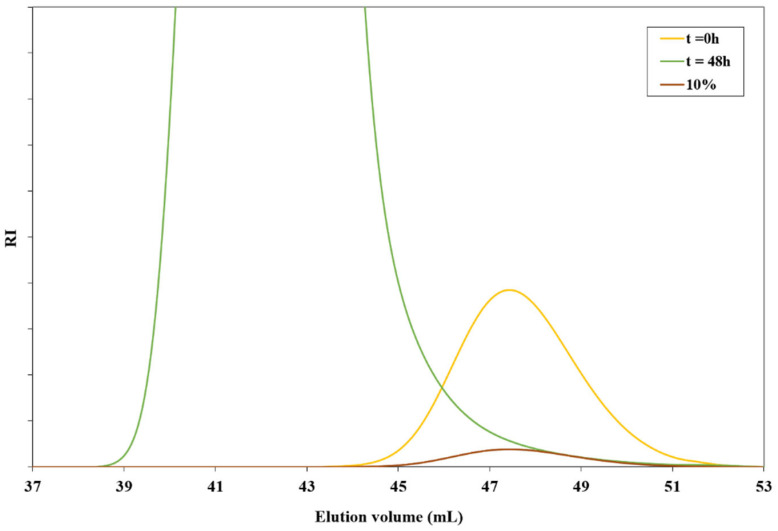
SEC chromatograms (RI detection).

**Figure A2:**
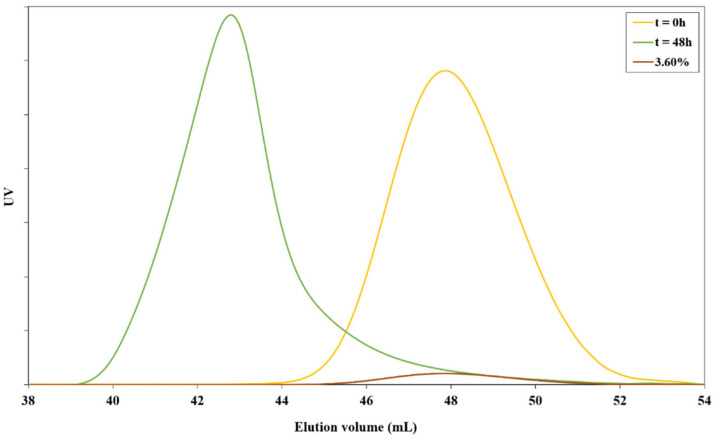
SEC chromatograms (UV detection).

## Data Availability

The data presented in this study are available on request from the corresponding author.

## Supplementary Materials

The following supporting information can be downloaded at: https://www.mdpi.com/article/10.3390/molecules29102174/s1, Figure S1: Mayo plot for the determination of chain transfer constant to xanthate **9** in styrene polymerization. Conditions of Table S1 (entries 1–4); Figure S2: Mayo plot for the determination of chain transfer constant to xanthate **10** in styrene polymerization. Conditions of Table S1 (entries 5–8); Figure S3: Mayo plot for the determination of chain transfer constant to xanthate **11** in styrene polymerization. Conditions of Table S1 (entries 9–12); Figure S4: Mayo plot for the determination of chain transfer constant to xanthate **13** in styrene polymerization. Conditions of Table S1 (entries 13–16); Figure S5: An example of SEC chromatograms during St polymerization in the presence of PSt-**14**; Table S1: Data used to determine transfer constants to xanthates C_tr_ (X) in RAFT/MADIX polymerization of styrene in bulk at 110 °C (self-initiation); Table S2: **13**- and **14**-mediated RAFT/MADIX polymerization of styrene at 110 °C (self-initiation). [St]_0_/[X]_0_ = 80.

## Appendix A

### Calculation of the Fraction of Dead Chains F_dead_ in PSt-14

In order to determine F_dead_, a large excess of St was reacted at 110 °C for 48 h in the presence of PSt-**14**. In Figure A1 and Figure A2, the yellow trace corresponds to starting PSt-**14**, the green signal is the produced PSt after chain extension and the brown trace is the unreacted PSt-**14** after peak deconvolution. The latter population is a combination of dead (terminated) chains and xanthate-terminated (unreacted chains). The RI trace in Figure A1 corresponds to both types of chains whereas in Figure A2, the UV trace (λ = 290 nm) corresponds to **14**-capped chains of the macro-CTA. Consequently, F_dead_ = 0.1 (RI) − 0.036 (UV) = 0.064.
