# Supplementary material for: α-Amido Trifluoromethyl Xanthates: A New Class of RAFT/MADIX Agents
Source: Molecules. 2024 May 7;29(10):2174. doi: 10.3390/molecules29102174 (PMC11123788; doi:10.3390/molecules29102174)

SUPPLEMENTARY MATERIAL

α-Amido Trifluoromethyl Xanthates: a New Class of RAFT/MADIX Agents

Mathias Destarac [1,2,*], Juliette Ruchmann-Sternchuss [2], Eric Van Gramberen,[2] Xavier Vila[3] and Samir Z. Zard [3,*]

[1] Laboratoire Softmat, Université deToulouse, CNRS UMR 5623, Université Toulouse III-Paul Sabatier Sabatier, 31062 Toulouse, France

[2] Rhodia Opérations, Centre de Recherches et Technologies d'Aubervilliers, 93308 Aubervilliers, France

[3] Laboratoire de Synthèse Organique associé au C.N.R.S., UMR 7652, Ecole Polytechnique, 91128 Palaiseau, France

* Correspondence: mathias.destarac@univ-tlse3.fr; samir.zard@polytechnique.edu.

Table S1. Data used to determine transfer constants to xanthates C_{tr} *(X)* in RAFT/MADIX polymerization of styrene in bulk at 110°C (self-initiation).

Entry	Xanthate	$[St]_0/[X]_0$	t (min)	Conv (%)	M_n (g/mol)	Đ
**1**		41	90	11.9	2060	2.10
**2**	**9**	80	90	10.1	3550	2.17
**3**		120	90	13.0	4680	2.26
**4**		197	90	12.1	6960	2.35
**5**		39	45	12.8	1700	1.85
**6**	**10**	76	60	8.4	2680	2.08
**7**		96	45	4.3	3560	2.24
**8**		151	45	7.9	4610	2.29
**9**		38	45	9.7	1980	1.85
**10**	**11**	74	60	11.1	3420	2.11
**11**		131	45	4.8	5420	2.21
**12**		160	45	8.1	6360	2.17
**13**		38	30	1.0	980	1.43
**14**	**13**	82	30	1.2	1730	1.68
**15**		120	30	1.3	2440	1.88
**16**		178	30	0.9	3800	2.10

Figure S1. Mayo plot for the determination of chain transfer constant to xanthate **9** in styrene polymerization. Conditions of Table S1 (entries 1-4).

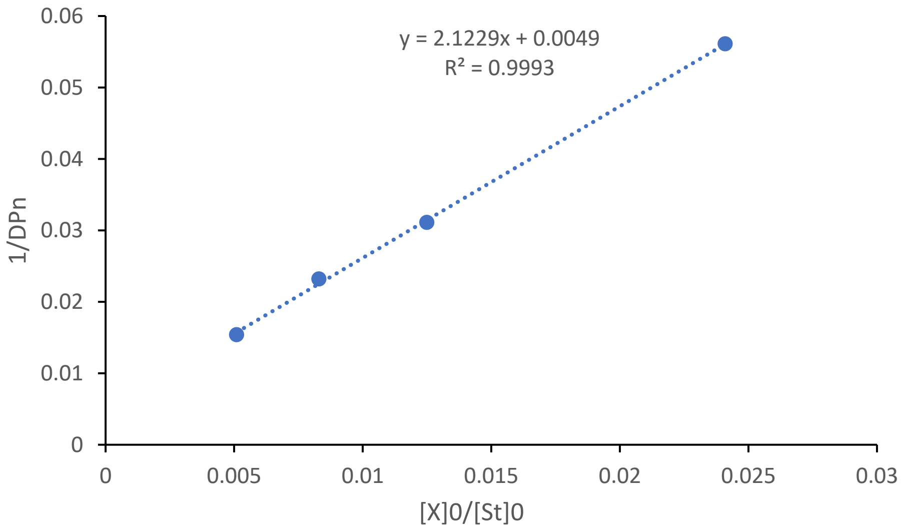

Figure S2. Mayo plot for the determination of chain transfer constant to xanthate **10** in styrene polymerization. Conditions of Table S1 (entries 5-8).

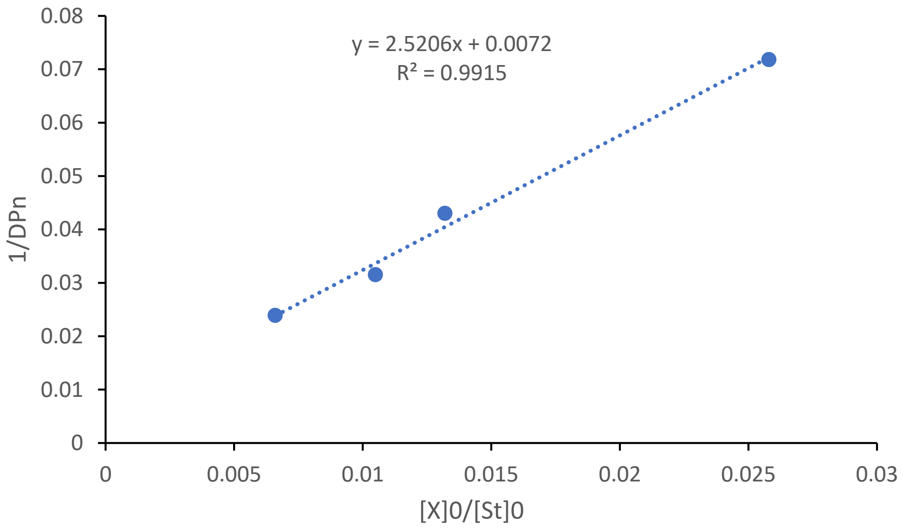

Figure S3. Mayo plot for the determination of chain transfer constant to xanthate **11** in styrene polymerization. Conditions of Table S1 (entries 9-12).

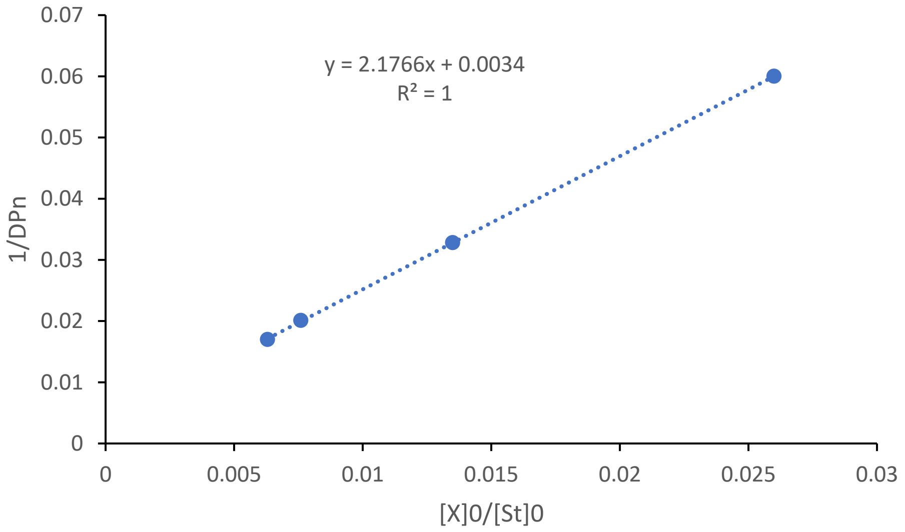

Figure S4. Mayo plot for the determination of chain transfer constant to xanthate **13** in styrene polymerization. Conditions of Table S1 (entries 13-16).

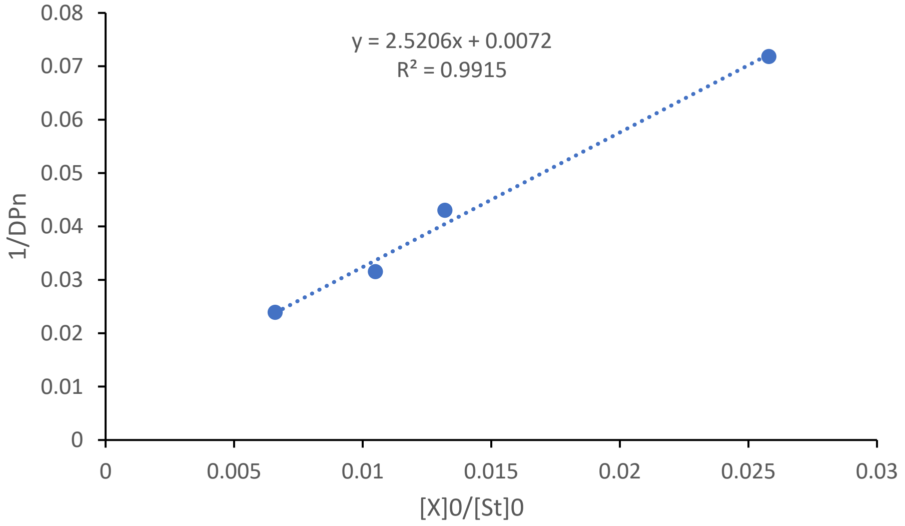

Table S2. 13- and **14**-mediated RAFT/MADIX polymerization of styrene at 110°C (self-initiation). $[St]_0/[X]_0$=80.

Entry	Xanthate	t (h)	Conv (%)	M_n (g/mol)	$M_{n\ th}$ (g/mol)	*Đ*
**1**		0.5	0.8	2060	387	1.69
**2**		1	3.4	3550	601	1.75
**3**	**13**	2	9.6	4680	1119	1.87
**4**		5	28.3	6960	2690	2.29
**5**		24	96.0	8220	8361	2.39
**6**		1	4.12	1190	658	1.69
**7**	**14**	2	12.1	1470	1323	1.80
**8**		5	25.8	2570	2461	1.92
**9**		24	85.0	6700	7393	1.49

Figure S5. An example of SEC chromatograms during St polymerization in the presence of PSt-**14**, for a given $[BPO]_0$ at 80°C. Plotting $Ln(S/S_0)$ as a function of time gives access to k_{act}.

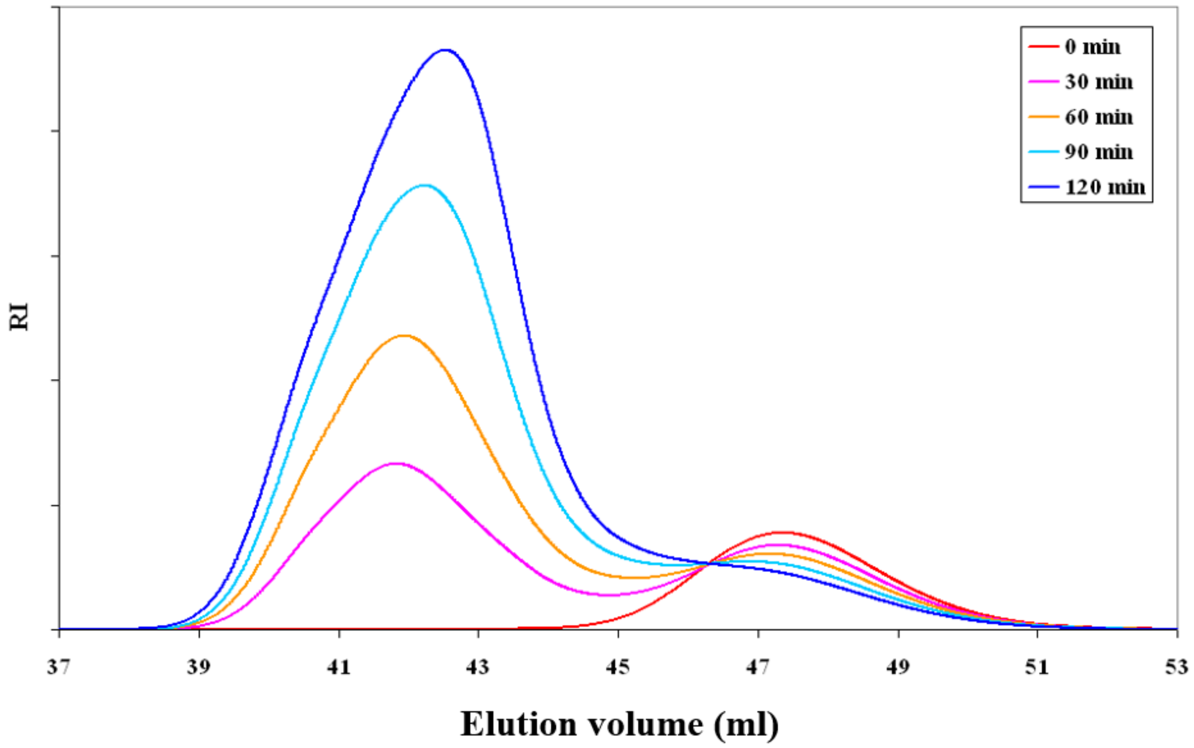

Supplement: Supplementary file 1 [file molecules-29-02174-s001.zip › molecules-2980981-supplementary.pdf]
